# Characteristics of lung cancer among patients with idiopathic pulmonary fibrosis and interstitial lung disease – analysis of institutional and population data

**DOI:** 10.1186/s12931-018-0899-4

**Published:** 2018-10-03

**Authors:** Joo Heung Yoon, Mehdi Nouraie, Xiaoping Chen, Richard H Zou, Jacobo Sellares, Kristen L Veraldi, Jared Chiarchiaro, Kathleen Lindell, David O Wilson, Naftali Kaminski, Timothy Burns, Humberto Trejo Bittar, Samuel Yousem, Kevin Gibson, Daniel J Kass

**Affiliations:** 1 Dorothy P. and Richard P. Simmons Center for Interstitial Lung Disease and Division of Pulmonary, Allergy, and Critical Care Medicine, University of Pittsburgh, NW 628 UPMC Montefiore, 3459 Fifth Avenue Pittsburgh, Pittsburgh, PA 15213 USA; 2 Interstitial Lung Diseases Program, Servei de Pneumologia, Institut Clinic Respiratori, Barcelona, Spain; 3 Section of Pulmonary, Critical Care and Sleep Medicine, Yale University, New Haven, CT USA; 4 Division of Hematology and Oncology, University of Pittsburgh, Pittsburgh, PA USA; 5 Department of Pathology, University of Pittsburgh, Pittsburgh, PA USA

**Keywords:** Idiopathic pulmonary fibrosis, Lung cancer

## Abstract

**Background:**

Lung Cancer is occasionally observed in patients with Idiopathic Pulmonary Fibrosis (IPF). We sought to describe the epidemiologic and clinical characteristics of lung cancer for patients with IPF and other interstitial lung disease (ILD) using institutional and statewide data registries.

**Methods:**

We conducted a retrospective analysis of IPF and non-IPF ILD patients from the ILD center registry, to compare with lung cancer registries at the University of Pittsburgh as well as with population data of lung cancer obtained from Pennsylvania Department of Health between 2000 and 2015.

**Results:**

Among 1108 IPF patients, 31 patients were identified with IPF and lung cancer. The age-adjusted standard incidence ratio of lung cancer was 3.34 (with IPF) and 2.3 (with non-IPF ILD) (between-group Hazard ratio = 1.4, *p* = 0.3). Lung cancer worsened the mortality of IPF (*p* <  0.001). Lung cancer with IPF had higher mortality compared to lung cancer in non-IPF ILD (Hazard ratio = 6.2, *p* = 0.001). Lung cancer among IPF was characterized by a predilection for lower lobes (63% vs. 26% in non-IPF lung cancer, *p* <  0.001) and by squamous cell histology (41% vs. 29%, *p* = 0.07). Increased incidence of lung cancer was observed among single lung transplant (SLT) recipients for IPF (13 out of 97, 13.4%), with increased mortality compared to SLT for IPF without lung cancer (*p* = 0.028) during observational period.

**Conclusions:**

Lung cancer is approximately 3.34 times more frequently diagnosed in IPF patients compared to general population, and associated with worse prognosis compared with IPF without lung cancer, with squamous cell carcinoma and lower lobe predilection. The causality between non-smoking IPF patients and lung cancer is to be determined.

**Electronic supplementary material:**

The online version of this article (10.1186/s12931-018-0899-4) contains supplementary material, which is available to authorized users.

## Background

Idiopathic pulmonary fibrosis (IPF) is a fatal pulmonary condition characterized by the accumulation of activated fibroblasts and extracellular matrix within the lung parenchyma that exhibits progressive, but unpredictable disease course [1, 2]. Two medications are currently approved for the treatment of IPF, but their effects on mortality and quality-of-life in IPF are uncertain [3, 4]. IPF is the most common type of the idiopathic interstitial pneumonias [5], and its distinctive radiographic and pathologic characteristics, as well as course of disease differentiate it from other types of interstitial lung disease (ILD) [6, 7]. Although the link between IPF and lung cancer has been known for years [8], estimates of the prevalence of lung cancer among IPF have varied widely [9, 10]. The effect of a lung cancer diagnosis on the prognosis of IPF is also an unsettled question. Some have suggested IPF and lung cancer exhibit no differences in survival rate compared to IPF without lung cancer [11–14], but a more recent study has supported a worse prognosis [15].

In this context, the purpose of our study was to compare primary lung cancers in a large cohort of patients with IPF and non-IPF ILD to lung cancers from population data in order to describe the characteristics of and to estimate the incidence and prevalence of lung cancer in patients with IPF and non-IPF ILD.

## Methods

### Regulatory approval

The ILD registry at the Dorothy P. and Richard P. Simmons Center for Interstitial Lung Disease and the Lung Cancer registry at the Hillman Cancer Center were approved by the Institutional Review Board at the University of Pittsburgh.

### Registries data collection

**Fig. 1:**
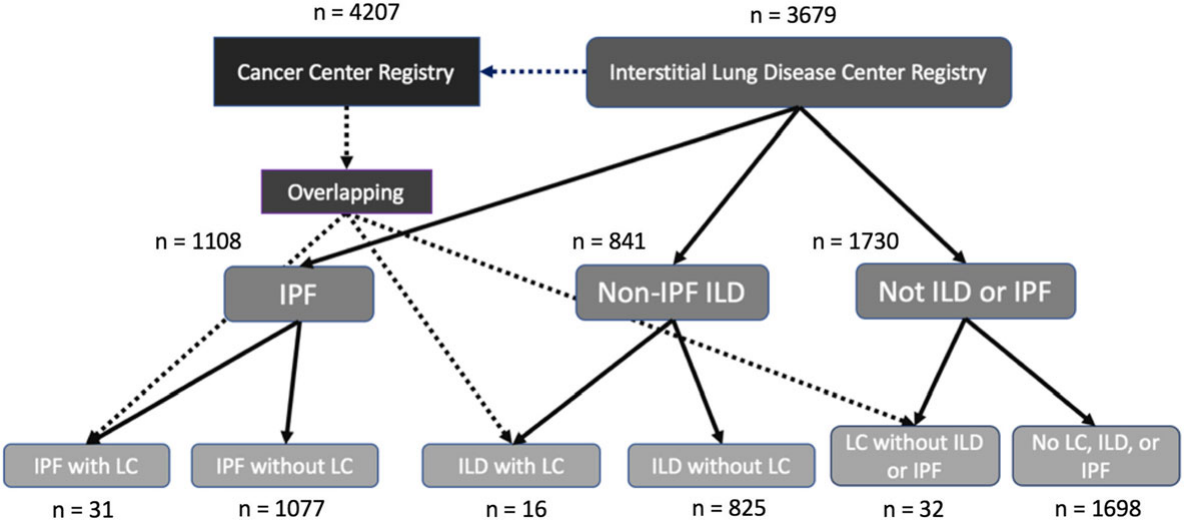
Matching of Interstitial Lung Disease (ILD) and Cancer Center Datasets Data from the Simmons Center for ILD were cross-matched with Lung Cancer data from the Hillman Cancer Center to identify patients with and without lung cancer, Idiopathic Pulmonary Fibrosis (IPF), or non-IPF ILD. *Definition of abbreviations*: ILD = interstitial lung disease; IPF = idiopathic pulmonary fibrosis; LC = lung cancer

Data from the Simmons Center for Interstitial Lung Disease were collected from January 2000 to December 2015. The Simmons registry diagnosis was based on established American Thoracic Society and European Respiratory Society clinical criteria [1, 16]. For lung cancer, registry data were obtained from the Hillman Cancer Center during the same observation period. Both registries are linked to providers’ clinics, and are actively followed, and vital status is regularly updated from the Social Security Database. Patients from the ILD registry were characterized as having IPF or non-IPF ILD. Non-IPF ILD refers to ILD caused by a disease other than IPF. The combination of the two registries (referred as ‘institutional data’) according to each disease status of IPF, non-IPF ILD, and lung cancer is shown in Fig. 1. To obtain the radiographic evidence of emphysema among the two institutional registries, natural language process was used to extract keywords including ‘emphysema’ or ‘emphysematous’, with its positive and negative terms from the chest computed tomography (CT) radiology reports.

### Population data collection

Epidemiologic data of new lung cancer cases were obtained from the Cancer Registry of the Department of Health of the Commonwealth of Pennsylvania, which is a part of the National Program of Cancer Registry. The following queries were used: (1) demographic data: age, gender, ethnicity; (2) Clinical data: age at lung cancer diagnosis, cancer stage according to American Joint Committee on Cancer staging guidelines [17, 18], histology, location, and mortality. The presence of IPF or non-IPF ILD was not identifiable from this data, as they were not collected in this epidemiologic registry. Age-related State Census data was obtained from the Enterprise Data Dissemination Informatics Exchange (EDDIE) for calculating population cancer statistics (http://www.statistics.health.pa.gov/StatisticalResources/EDDIE, last accessed May 15, 2017).

### Data management

To extract the data, a standard application programming interface, Microsoft Open Database Connectivity (Microsoft Corporation, Redmond, WA) was used. Lung cancers were classified following the World Health Organization classification [19]. TNM system was used to stage lung cancers at the time of diagnosis. Diagnosis of IPF and other ILDs, as well as lung cancer were based on the codebook of International Classification of Disease, Ninth Edition (ICD-9) at the time of diagnosis. Time of the diagnosis for IPF as well as non-IPF ILD was defined by the first ILD center visit when the ICD-9 code was given. Age-adjusted standard incidence ratio of lung cancer in IPF and non-IPF ILD patients were compared with the DOH data. For the calculation of the survival rate, the time from the time of diagnosis of IPF or non-IPF ILD to death (survival time) were compared. The location of lung cancers, the histologic phenotypes, and the stage at diagnosis were compared.

### Statistical analysis

Continuous and categorical variables were compared between different groups using the Kruskal-Wallis or Fisher’s exact test. Log rank test was performed to compare the survival function in lung cancer patients with and without IPF. Standardized incidence ratio was used to compare age-adjusted incidence of lung cancer between IPF patient and lung cancer registry information from Pennsylvania DOH. Age-adjusted standardized incidence ratio was calculated defining each expected and observed cases for group is based on person-years. The person-year was calculated by the sum of the time between the date of diagnosis of IPF or non-IPF ILD and either the date of death, or the end of the observation period (December 31, 2015) for each age bracket. All analyses were performed in STATA 14.2 (StataCorp., College Station, TX).

## Results

### Patient demographics and characteristics

We identified 1953 patients including 1108 IPF patients and 841 patients with non-IPF ILD diagnosis from the Simmons ILD data (Fig. 1). The baseline demographic profiles of institutional IPF patients were compared to non-IPF ILD patients (Table 1). When compared with non-IPF ILD group, IPF patients were older at diagnosis (median of 69 vs 59 years old), more frequently Caucasian (91% vs 85%), and male (59% vs 38%, all *p* < 0.001). The difference in prevalence of lung cancer between IPF and non-IPF ILD was not statistically significant (2.8 vs 1.9%, *p* = 0.12). More IPF patients than non-IPF ILD patients were smokers (66% vs 57%, *p* = 0.001). All-cause mortality was higher in IPF than non-IPF ILD group (47 vs 12%, *p* < 0.001). From the Hillman Cancer Center Registry, 4207 patients were identified for review for lung cancer of all histologic subtypes including 4176 cases without IPF diagnosis. Following cross-matching of the two registries, we found 31 patients with both IPF and lung cancer and 16 patients with non-IPF ILD and lung cancer. Primary etiologies included in non-IPF ILD group are summarized in Additional file 1: Table S1.

**Table 1:** Baseline demographics and clinical characteristics of IPF and non-IPF ILD from institutional registry

Variables	IPF		Non-IPF ILD		*p*-values
	patients	Results	patients	Results	
Age at diagnosis (years)	1108	69 (62–75)	841	59 (50–68)	< 0.001
Gender (% male)	1108	652 (59%)	841	318 (38%)	< 0.001
Ethnicity (% Caucasian)	1108	1007 (91%)	841	712 (85%)	< 0.001
Smoking – Never	1054	361 (34%)	443	193 (44%)	< 0.001
Former		655 (62%)		224 (51%)	
Current		38 (4%)		26 (6%)	
Prevalence of lung cancer	1108	31 (2.8%)	841	16 (1.9%)	0.20
Mortality over observation period (%)	1108	515 (47%)	841	104 (12%)	< 0.001

*Definition of abbreviations*: *IPF* idiopathic pulmonary fibrosis, *ILD* interstitial lung disease. Mortality based on patient records as deceased at the time of the review of the registry data

The demographic characteristics of patients with lung cancer and IPF were compared to lung cancer without IPF from the Hillman Cancer Center Registry, as well as with lung cancer from the population data acquired from Department of Health (DOH) (Table 2). From the DOH data, the total obtained number of lung cancer cases from 2000 to 2015 was 156,032. After excluding duplicated identifiers (*n* = 3951) and non-solid and non-primary (either metastatic disease from other primaries or unknown primary) (*n* = 47,127), 104,954 patients were included in the final analysis.

**Table 2:** Baseline demographics of lung cancer patients with and without idiopathic pulmonary fibrosis (IPF) from institution, and lung cancer in overall population of state of Pennsylvania

Variables	Lung cancer with IPF		Lung cancer without IPF (institutional data)		Lung cancer without IPF (population data)		*p*-values column 1 vs. 3
	N	Results	N	Results	N	Results	
Age in years, median (IQR)	31	65 (62–71)	4176	67 (60–75)	104,954	69 (61–77)	0.03
Gender (% male)	31	19 (61%)	4176	2023 (48%)	104,954	53,800 (51%)	0.16
Ethnicity (% Caucasian)	31	30 (97%)	4176	3679 (88%)	104,954	89,643 (85%)	0.07
Smoking – never	31	7 (23%)	4176	395 (10%)	NA		< 0.001 (column 1 vs 2)
former		24 (77%)		2362 (57%)			
current		0		1361 (33%)			
Mortality over observation period (%)	31	26 (84%)	4176	2644 (63%)	104,954	78,254 (75%)	0.2
Survival time in months, median (IQR)	26	5 (1–10)	4176	13 (6–26)	NA	NA	0.002 (column 1 vs 2)

*Definition of abbreviations*: *IQR* Interquartile range. Column 1 = Lung cancer with IPF; column 2 = Lung cancer with non-IPF ILD; column 3 = Lung cancer in general population in Pennsylvania. *NA* not available. Survival time was calculated by the time from lung cancer diagnosis to the date of expiration, or the time of registry data review

IPF patients with lung cancer were not significantly different in terms of age (65 vs 67 years, *p* = 0.2), gender (61 vs 48% male, *p* = 0.15), and race (97 vs 88% Caucasian, *p* = 0.14) compared to lung cancer patients without IPF from the Hillman Cancer Center Registry. Fewer IPF patients with lung cancer were former or current smokers compared to lung cancer patients without IPF (77 vs. 90%, *p* < 0.001), with 7 never-smokers (23%) were identified from IPF and lung cancer group. No smoking history data were available from the Pennsylvania Department of Health database. IPF patients with lung cancer were, however, younger compared to the population data for lung cancer (65 vs 69 years, *p* = 0.03). There was no difference in the gender and race between the ILD data and population data. The mortality between lung cancer with IPF and lung cancer without IPF was different when compared within the institution (84 vs 63%, *p* = 0.013). However, this difference was not observed when institutional IPF patients with co-morbid lung cancer were compared to population data (84 vs 75%, *p* = 0.2) over the 15 years of observational period. To better understand the prognosis of IPF with lung cancer, we examined survival time as defined as time interval from the date of lung cancer diagnosis to the date of death. In the institutional cohort, patients with lung cancer without IPF had a significantly longer survival time, compared to lung cancer patients with IPF (median survival time 5 vs 13 months, *p* = 0.002).

### Cumulative incidence of lung cancer in IPF and non-IPF ILD

**Fig. 2:**
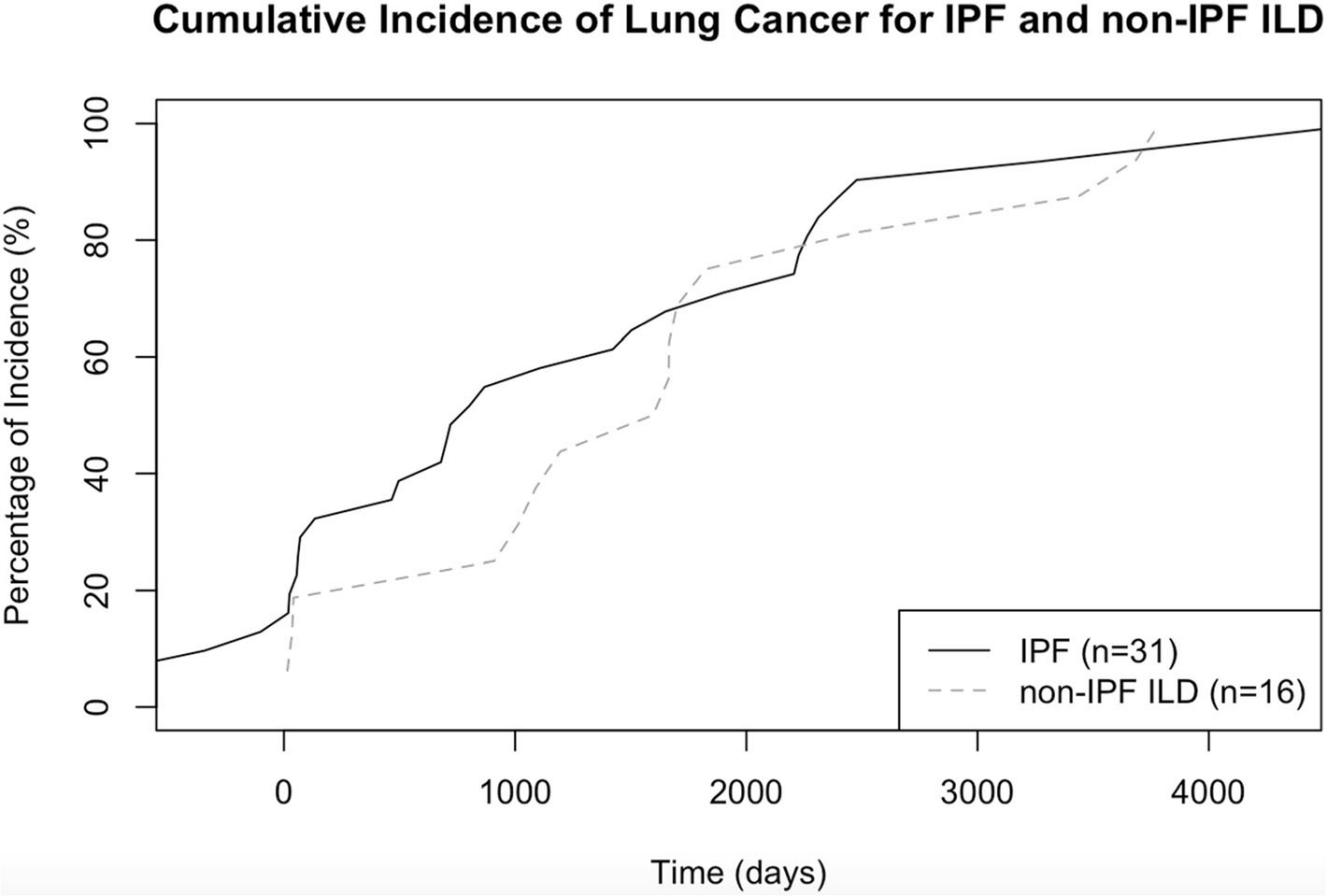
Cumulative incidence of lung cancer among IPF (*solid, n = 31*) and non-IPF ILD patients (*hatched, n = 16*). *Definition of abbreviations*: ILD = interstitial lung disease; IPF = idiopathic pulmonary fibrosis

We next examined the cumulative incidence of lung cancer in IPF and non-IPF ILD patients. The median time to the discovery of lung cancer after the diagnosis of IPF was 53 months (interquartile range (IQR) 25–77 months), and 55 months (IQR 44–62 months) in non-IPF ILD. Although there was no observed difference in the median time to the discovery of lung cancer between these groups, there did appear to be increased incidence of lung cancer in the first 2 years after diagnosis in the IPF group which persisted until year four (Fig. 2).

### Age-adjusted incidence of lung cancer among IPF compared to general population in Pennsylvania

Observed incidence of lung cancer (Table 3) in general population was calculated within different age brackets, and showed that the rate among IPF patients was 3.34 times higher (95% confidence interval 2.3–4.7) and in non-IPF ILD patients 2.3 times higher (95%CI 1.3–3.6) compared to general population. To drill down the cancer risk in non-IPF ILD cases, the non-IPF ILD group was subdivided further into ILD associated with autoimmunity, hypersensitivity pneumonitis (HP), pneumoconiosis, and smoking-related ILDs (eosinophilic granuloma, respiratory bronchiolitis-associated interstitial lung disease, and desquamative interstitial pneumonia). No lung cancers were observed in the HP or pneumoconiosis groups. Among non-IPF ILD patients, 508 patients with ILD related to autoimmunity (including systemic sclerosis, rheumatoid arthritis, polymyositis/dermatomyositis, Sjogren’s disease, and mixed connective tissue disease) were identified, among them 12 lung cancer cases diagnosed within the observation period. When the autoimmunity group is considered alone, the age-adjusted incidence of lung cancer was 4.95 times higher than general population (95% CI 2.7–8.4). The rate of non-smokers among autoimmune-related ILD patients were 47% (123 out of 261 known history of smoking). Among patients with HP (*n* = 109), no lung cancer was identified, in which 48% (28 out of 58 known history of smoking) were smokers. From a small group of patients (*n* = 47) with smoking-related ILDs, two cases of lung cancer were found.

**Table 3:** Age-adjusted standardized incidence rate of Idiopathic pulmonary fibrosis (IPF), and non-IPF interstitial lung disease (ILD) and lung cancer from combined registries, compared to Department of Health lung cancer registry and Pennsylvania census data, based on 1000 person-year for each expected and observed age group. Observed incidence of lung cancer in general population was calculated within different age brackets, as well as lung cancer occurrence in IPF and ILD population

	Registry IPF and LC over population	Registry non-IPF ILD and LC over population
Case number	Observed / Expected cases	Observed / Expected cases
	31 / 9.28	16 / 7.05
SIR (95% CI)	3.34 (2.31–4.68)	2.27 (1.34–3.61)

*Definition of abbreviations*: *SIR* standardized incidence ratio, *CI* confidence interval, *IPF* idiopathic pulmonary fibrosis, *LC* lung cancer

### Survival estimates of lung cancer in IPF

**Fig. 3:**
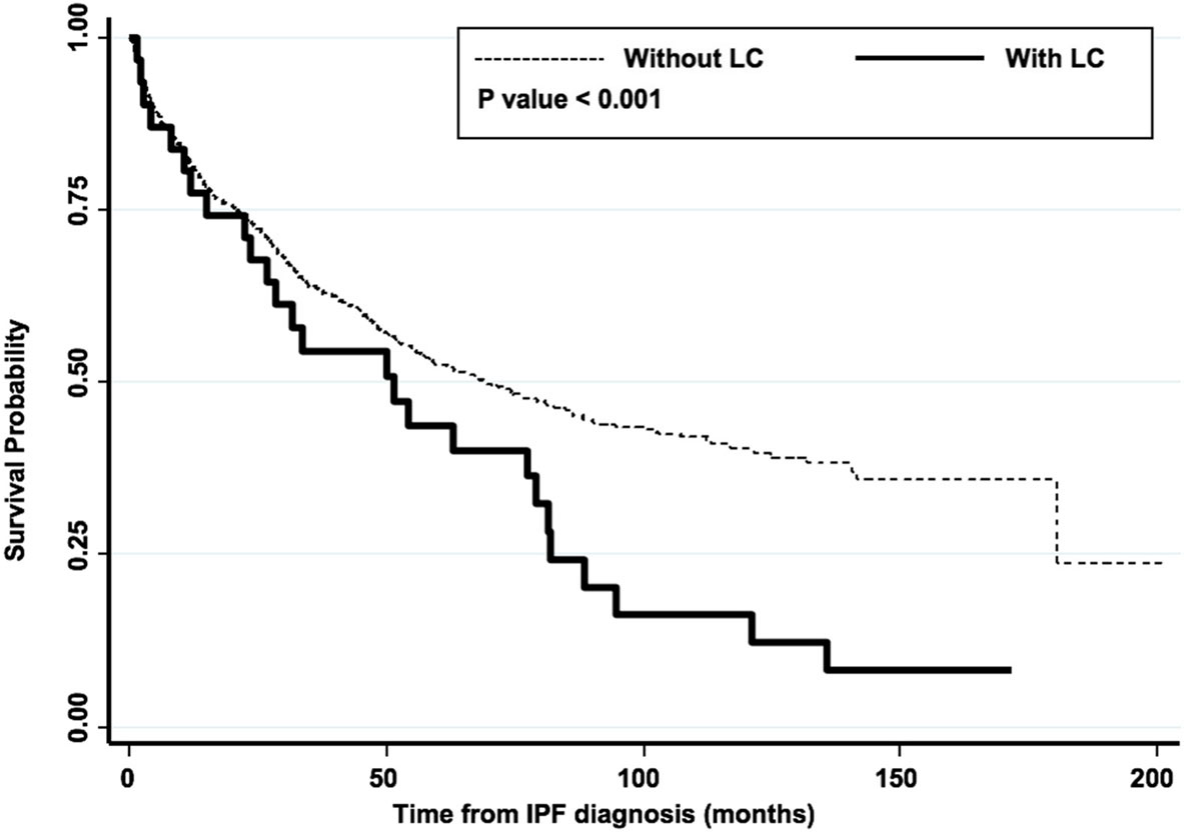
Comparison of survival probabilities of IPF patients with and without co-morbid lung cancer (between the two institutional datasets). *Definition of abbreviations*: IPF = idiopathic pulmonary fibrosis; LC = lung cancer

The survival probabilities for IPF without lung cancer (*n* = 1077) and IPF with lung cancer (*n* = 31) were compared over time. Figure 3 illustrates Kaplan-Meier curves for institutional data - IPF without lung cancer and IPF with lung cancer groups, where statistically significant (*p* < 0.001) lower survival rate over time was observed for IPF and lung cancer patients compared to IPF patients without lung cancer. The trend in cumulative incidences between the two groups showed no major differences across 15 years of accumulated follow-up period of variable lengths of observed individual cases.

### Clinical characteristic of lung cancer patients with IPF compared to non-IPF ILD and the Department of Health data

The clinical characteristics of lung cancer with a diagnosis of IPF and non-IPF ILD are described in Table 4. The primary site of the lung cancer for IPF patients showed more frequent lower lobe predilection compared to population lung cancer data (63% vs. 36%, *p* < 0.001). On the other hand, lung cancer diagnosed from institutional non-IPF ILD patients demonstrated no statistically significant differences in location compared to population lung cancer data (*p* = 0.2). The most common type of lung cancer in IPF patient to be squamous cell cancer, higher in IPF patients (41%) compared to non-IPF ILD patients (19%) with lung cancer within the institution. Squamous cancer in IPF was also more common compared with squamous cell cancer ratio in population data (29%) but not reaching statistical significance (*p* = 0.07). The histology of lung cancer was not significantly different between non-IPF ILD patients and general population (*p* = 0.4). No significant differences in distribution of lung cancer stage in IPF compared to patients with lung cancer without IPF were identified in the DOH data (*p* = 0.6). The similarity in the cancer stage distribution also exists when compared with the institutional non-IPF ILD patients with lung cancer data (*p* = 0.8), nor with the population data (*p* = 0.6). Lung cancer patients with non-IPF ILD also demonstrated no differences compared to population data. Individual level descriptive analysis for all 31 patients diagnosed with IPF and lung cancer are available (Additional file 1: Table S2**)**.

**Table 4:** Characteristics of lung cancer in patients with IPF or non-IPF ILD compared to Pennsylvania state data

Variables	Lung cancer with IPF		Lung cancer with non-IPF ILD		Lung cancer in general population in Pennsylvania		*p*-value (column 1 vs. 3)
	N	Results	N	Results	N	Results	
Laterality	31		16		100,949		0.4
Left		14 (45%)		7 (44%)		41,745 (41%)	
Right		16 (52%)		7 (44%)		57,566 (57%)	
Bilateral		1 (3%)		2 (12%)		1638 (2%)	
Primary site	27		13		85,087		< 0.001
Upper lobe		8 (27%)		6 (46%)		53,288 (63%)	
Lower lobe		17 (63%)		6 (46%)		30,422 (36%)	
Others		2 (7%)		1 (8%)		1377 (2%)	
Histology	27		16		104,954		0.07
SCC		11 (41%)		3 (19%)		30,596 (29%)	
Adenocarcinoma		7 (26%)		10 (63%)		49,217 (47%)	
Other		9 (33%)		3 (19%)		25,141 (24%)	
Stage	29		16		83,994		0.6
I		9 (31%)		4 (25%)		19,000 (23%)	
II		2 (7%)		1 (6%)		4312 (5%)	
III		7 (24%)		6 (38%)		21,730 (26%)	
IV		11 (38%)		5 (31%)		38,952 (46%)	

*Definition of abbreviations*: *SCC* squamous cell cancer, *NA* not available. Column 1 = Lung cancer with IPF; column 2 = Lung cancer with non-IPF ILD; column 3 = Lung cancer in general population in Pennsylvania

Among our 1108 IPF patients, 233 (21%) cases were found to have emphysema as a comorbid condition during the clinic encounters. From 841 non-IPF ILD patients, 156 (19%) had a diagnosis of emphysema. For the patients diagnosed with lung cancer along with IPF, 17 patients had a diagnosis of emphysema. We observed that radiologic emphysema was overrepresented in IPF patients with lung cancer (OR 5.2) compared to non-IPF patients from Hillman Cancer Center data.

Transplantation and immunosuppression have been associated with an increased rate of cancer development. This is especially true as single lung transplant has been known for higher rate of lung cancer in native lung, ranging from 6.9–9.8% [20, 21]. To estimate the effect of single lung transplant on development of lung cancer for IPF patients, a subgroup analysis was performed (Additional file 1: Table S**3**) within the institutional registry data. With the data collected until 2014, a total of 97 IPF with single lung transplanted patients were enrolled in our registry. Among them, 13 cases of lung cancer were identified in the native lung (13.4%) following single lung transplant (SLT). There was no difference in age, gender, ethnicity, or smoking history, but the mortality of single lung transplant recipients who developed lung cancer (12 out of 13 patients, 92%) were significantly higher than single lung transplant without lung cancer (49 out of 84, 59%, *p* = 0.028) during follow-up period.

## Discussion

The association between IPF and lung cancer has been observed for years, although estimates of the risk have varied widely. Co-morbid lung cancer represents a very challenging problem for the clinician. In this study, we sought to identify the clinical characteristics of lung cancer among patients with IPF and non-IPF ILD from our institutional data and to compare these data from population data. In our study, which represents the largest review in the United States, we have found that patients with IPF exhibited a 3.34-fold higher incidence of lung cancer compared to the general population who developed lung cancer. IPF patients showed a statistically significant worse mortality rate over observation period compared to lung cancer patients without IPF. Consistent with several previous studies, lung cancer in IPF was observed more frequently in the lower lobes, with a borderline association between IPF and the squamous cell carcinoma histology, as observed previously [22–25]. Overall, our data suggest that lung cancer in IPF is phenotypically distinctive from “sporadic” lung cancer, and exhibits worse prognosis compared to IPF without lung cancer, or lung cancer without IPF.

We found that single lung transplant patients from IPF exhibited a higher prevalence of lung cancer, and the development of lung cancer in this group was associated with higher mortality during follow-up period. When the native IPF lung is exposed to immunosuppressive medications to prevent rejection from lung transplant, the risk of cancer may be accentuated by loss of immune-mediated “tumor surveillance” [20]. This raises the question if single lung transplant represents an additional risk factor for the development of lung cancer in IPF. While our numbers are small, the suggestion is that indeed, transplant enhances the risk of lung cancer. Furthermore, it begs the highly speculative question if IPF patients should undergo double lung transplant as a potential “cure” to the lung cancer risk. Additional study of single lung transplant for all indications might help elucidate the specific risk of lung cancer in IPF associated with immunosuppression.

The subject of IPF and lung cancer has been studied in diverse ethnic groups and health care systems. One of the largest studies of health care databases identified a relative risk 7.3 for lung cancer in IPF patients in the United Kingdom (UK) [12], which was validated by analysis of an overlapping UK database where 1064 IPF patients were studied identifying a nearly fivefold increased rate of lung cancer [9]. Our estimate for incidence of lung cancer among IPF was lower at 3.3, showing little change after controlling for smoking in the British study [12]. While smoking rates were similar in the UK studies between IPF and control patients, smoking was less common in IPF patients with lung cancer compared to patients with lung cancer alone in our institutional data. These data corroborate the idea that IPF is a risk factor for lung cancer independent of smoking. It is possible that smoking may account for the observed prevalence of lung cancer in IPF of 13% in an Italian cohort [15], which is significantly higher than our observed prevalence of 2.8%. The number of never smokers in our cohort is at least three-fold higher than the Italian cohort. Another UK study cohort [26] showed that most IPF patients with lung cancer diagnosed had smoking histories (never smoker *n* = 2, 4.5%), along with other IPF and lung cancer studies [13, 27, 28]. In our analysis, the IPF patients without a history of smoking did not exhibit an increased risk of lung cancer, which suggests that cigarette smoking could be an important contributor of lung cancer development. The role of genetics in IPF could play a pivotal role in the development of lung cancer [29], but the mechanistic pathway has not been fully elucidated. Another factor that could drive the prevalence of lung cancer in pulmonary fibrosis cohort would be combined pulmonary fibrosis with emphysema (CPFE) [30]. Despite the higher lung cancer rate for IPF patients with radiologic emphysema (OR 5.2), the distribution of radiologic diagnosis of emphysema varied from 12 years before to 3 months after the diagnosis of lung cancer, and definitive quantification of impact of emphysema was hard to estimate from our results. In addition, concern of surveillance bias exists in our data because of the frequency of chest CTs in this population. Further study is needed to determine a dose-response effect for lung cancer risk in IPF possibly based on volumetric CT analysis.

Cancer risk in the non-IPF ILD population is well known. Some phenotypes of non-IPF ILD including polymyositis/dermatomyositis [31], rheumatoid arthritis [32], and systemic sclerosis [33] are known to be associated with increased risk for lung cancer. We found a prevalence of lung cancer in 1.9% among non-IPF ILD patients. This appears to be lower than some previously published estimates [34, 35], as well as a recent comprehensive study [36]. Thus, the molecular underpinnings of lung cancer risk in non-IPF ILD may be different than in IPF. Among non-IPF ILD patients, our analysis showed 4.95-fold increased age-adjusted incident rate of lung cancer among autoimmune disease-related ILD within the observation period. However, it is difficult to determine if the increased risk of lung cancer is associated with an autoimmune process versus immunosuppressive therapy which is needed to treat autoimmune disease. Further study is needed to quantify these risks at both the population level and at the molecular level.

Previous studies have suggested that mortality of patients with lung cancer and IPF was no greater than for IPF patients without lung cancer [23, 37]. More recent studies, however, have argued there that the mortality of lung cancer in IPF patients is higher than for lung cancer alone [15, 25] and that the presence of IPF is associated with a prognosis worse than lung cancer. We found that lung cancer worsened the prognosis of IPF. Based on the Simmons data and DOH data, however, having IPF did not increase the overall mortality of lung cancer in the general population in a statistically significant way. What explains this discrepancy? This observation may be influenced by other important prognostic information in the general population that are not available to us for study such as smoking status. The clinically relevant question here would be whether a screening process for lung cancer among IPF patients would yield survival benefit. However, there are no current recommendations to guide clinicians how to diagnose and manage lung cancer in IPF. It is unclear how treatment decisions should be made at an individual level. Does impaired pulmonary function affect treatment decisions? Stage at diagnosis did not differ between IPF and non IPF patients. These data highlight many of the unanswered questions surrounding lung cancer and IPF.

Recently, the anti-cancer effect of IPF therapy has been suggested in recent studies [38–40]. This could raise an interesting question, whether the risk of lung cancer could be affected for our registry population with the use of pirfenidone. However, because IPF therapy was officially approved by the United States Food and Drug Administration in November 2014, and most of our patients’ data were collected beforehand, we cannot determine if therapy changes lung cancer risk in IPF. Thus, the direct effect of pirfenidone in our registry data on the development cannot be assessed in this study.

### Limitations

We recognize that there are several limitations to this study. This single-center study with retrospective database review may underrepresent the IPF and lung cancer population. Conversely, referral bias may inflate the prevalence as sicker and more complicated patients seek care at the tertiary specialty center. Surveillance bias for lung cancer could exist, with IPF or non-IPF ILD patients tend to visit clinic more often for diagnostic work-up or symptoms, while early-stage lung cancers may be asymptomatic. Evolving diagnostic criteria for IPF and ILD over the last 15 years may be associated with misclassification, with no clear unifying interpretation present for the changing criteria [41]. These factors may have potentially affected our analysis on the prevalence and survival trajectories because the possibility of misclassification over time. Third, some of our referrals were from out-of-state and might not be represented by the PA population data. The population data for lung cancer obtained from the Pennsylvania Department of Health lack smoking history and the exact time of lung cancer diagnosis (data only has year of diagnosis). For these reasons, assessment of risk factors for lung cancer and head-to-head survival analysis between institutional and the DOH data were not possible in this study.

## Conclusions

The incidence of lung cancer was higher among IPF patients compared to general population or ILD patients. Lung cancers in IPF was observed more commonly in never smokers and demonstrated predilection for the lower lobe, and the squamous cell histology may predominate. In addition, lung cancer negatively impacts the prognosis of IPF. Further study is needed to elucidate how the IPF phenotype alters the lung cancer phenotype and if screening for lung cancer in this population will ultimately impact the course of the disease.

## Additional file

Additional file 1: **Table S1.** A summary of the primary etiologies included in non-IPF ILD group. **Table S2.** Individual-level descriptive analysis for 31 consecutive patients diagnosed with lung cancer after diagnosis of IPF. **Table S3.** Demographic characteristics of lung cancer (LC) among single lung transplant (SLT) recipients among idiopathic pulmonary fibrosis (IPF) patients. (DOCX 23 kb)
