# Supplementary material for: Characteristics of lung cancer among patients with idiopathic pulmonary fibrosis and interstitial lung disease – analysis of institutional and population data
Source: Respir Res. 2018 Oct 3;19:195. doi: 10.1186/s12931-018-0899-4 (PMC6171146; doi:10.1186/s12931-018-0899-4)
Supplement: Supplementary file 1 — Table S1. A summary of the primary etiologies included in non-IPF ILD group. Table S2. Individual-level descriptive analysis for 31 consecutive patients diagnosed with lung cancer after diagnosis of IPF. Table S3. Demographic characteristics of lung cancer (LC) among single lung transplant (SLT) recipients among idiopathic pulmonary fibrosis (IPF) patients. (DOCX 23 kb) [file 12931_2018_899_MOESM1_ESM.docx]

Characteristics of lung cancer among idiopathic pulmonary fibrosis and interstitial lung disease patients - institutional and population data analysis

Supplementary Materials

Additional file 1: **Table S1. A summary of the primary etiologies included in non-IPF ILD group**

| **Primary Etiology** | **Number of Cases** |
| --- | --- |
| **Systemic Sclerosis** | **175** |
| **Hypersensitivity pneumonitis** | **109** |
| **Mixed connective tissue disease** | **95** |
| **Rheumatoid arthritis** | **68** |
| **Sjogren’s disease** | **67** |
| **Polymyositis/Dermatomyositis** | **56** |
| **Anti-Synthetase syndrome** | **30** |
| **Post-inflammatory** | **25** |
| **Eosinophilic granuloma** | **22** |
| **NSIP (cellular + fibrotic)** | **21** |
| **Asbestosis** | **17** |
| **Respiratory bronchiolitis-Interstitial Lung Disease** | **26** |
| **Chronic aspiration** | **15** |
| **Silicosis** | **14** |
| **Chemotherapy/drug-induced** | **12** |
| **Connective-tissue disease related (without further classification)** | **10** |
| **Chronic eosinophilic pneumonia** | **9** |
| **Systemic lupus erythematosus** | **5** |
| **Radiation-induced** | **4** |
| **Sarcoidosis** | **4** |
| **Lymphoid interstitial pneumonitis** | **3** |
| **Microscopic polyangiitis** | **2** |
| **Bronchiolocentric interstitial pneumonia** | **2** |
| **Talc** | **1** |
| **Not fully characterized** | **49** |
| **TOTAL** | **841** |

*Definition of abbreviations*: NSIP = Nonspecific interstitial pneumonitis; ILD = interstitial lung disease; IPF = idiopathic pulmonary fibrosis

Additional file 1: **Table S2. Individual-level descriptive analysis for 31 consecutive patients diagnosed with lung cancer after diagnosis of IPF.**

| Patient number | Age at Dx | Gender | Histology | Stage at Dx | Days survived |
| --- | --- | --- | --- | --- | --- |
| 1 | 65 | M | SCLC | 4 | 22 |
| 2 | 39 | F | Adeno | 4 | 160 |
| 3 | 61 | F | Adeno | 2A | 987 |
| 4 | 74 | F | Squamous | 4 | 142 |
| 5 | 66 | F | NEC | NA | 435 |
| 6 | 69 | M | Squamous | 4 | 105 |
| 7 | 58 | M | Squamous | 4 | 99 |
| 8 | 68 | M | Squamous | 3A | 212 |
| 9 | 75 | M | Squamous | 1A | 211 |
| 10 | 71 | M | SCLC | 4 | 16 |
| 11 | 66 | M | Squamous | 2B | 4 |
| 12 | 63 | F | SCLC | 4 | 143 |
| 13 | 67 | M | Adeno | 3A | 257 |
| 14 | 79 | F | Squamous | 1B | 408 |
| 15 | 67 | M | SCLC | NA | 220 |
| 16 | 61 | F | Adeno | 1A | 17 |
| 17 | 71 | M | Adeno | 4 | 4 |
| 18 | 60 | M | Sarcoma | 1B | 188 |
| 19 | 77 | M | Squamous | 1B | 204 |
| 20 | 71 | F | Adeno | 4 | 300 |
| 21 | 82 | M | SCLC | NA | 147 |
| 22 | 68 | M | SCLC | 3A | 30 |
| 23 | 74 | F | Adeno | 1B | 146 |
| 24 | 64 | F | Adeno | 3A | 361 |
| 25 | 65 | M | Squamous | 1A | 350 |
| 26 | 70 | F | Adeno | 1B | 2553 |
| 27 | 67 | M | Adeno | 4 | 13 |
| 28 | 80 | M | Squamous | 4 | 199 |
| 29 | 48 | F | NEC | 1A | 228 |
| 30 | 70 | M | Squamous | 3A | 15 |
| 31 | 71 | M | Squamous | 3A | 2073 |

*Definition of abbreviations*: M=male, F=female; SCLC=Small cell lung cancer, NEC=Neuroendocrine cell tumor; Adeno=adenocarcinoma; Squamous=squamous cell carcinoma; Dx = Diagnosis; Age at Dx = Age at both lung cancer and IPF diagnosis were established. d = days after both lung cancer and IPF diagnosis were established. Stage at diagnosis followed World Health Organization criteria as cited (1).

1. Travis WD, Brambilla E, Nicholson AG, Yatabe Y, Austin JH, Beasley MB, et al. The 2015 World Health Organization Classification of Lung Tumors: Impact of Genetic, Clinical and Radiologic Advances Since the 2004 Classification. Journal of thoracic oncology : official publication of the International Association for the Study of Lung Cancer. 2015;10(9):1243-60.

Additional file 1: **Table S3. Demographic characteristics of lung cancer (LC) among single lung transplant (SLT) recipients among idiopathic pulmonary fibrosis (IPF) patients.**

| Variables | IPF + SLT + LC | | IPF + SLT without LC | | p-values |
| --- | --- | --- | --- | --- | --- |
|  | patients | Results | patients | Results |  |
| Age (years) | 13 | 64 (62-68) | 84 | 64 (58-69) | 0.8 |
| Gender (% male) | 13 | 8 (62%) | 84 | 57 (68%) | 0.8 |
| Ethnicity (% Caucasian) | 13 | 13 (100%) | 84 | 79 (94%) | >0.9 |
| Smoking – Never | 13 | 5 (38%) | 81 | 26 (32%) | 0.8 |
| Former |  | 8 (62%) |  | 55 (68%) |  |
| Current |  | 0 |  | 0 |  |
| Mortality (%) | 13 | 12 (92%) | 84 | 50 (59%) | 0.028 |

*Definition of abbreviations*: IPF = idiopathic pulmonary fibrosis; SLT = single lung transplant; LC = lung cancer

**All single lung transplant done from 2000 to 2014 were included, with same observation window of 2000-2015, given the malignancies associated with transplant are most prevalent after 1 year of transplant.**
